# Method for Measurement of Viral Fusion Kinetics at the Single Particle Level

**DOI:** 10.3791/1484

**Published:** 2009-09-07

**Authors:** Daniel L. Floyd, Stephen C. Harrison, Antoine M. van Oijen

**Affiliations:** Department of Biological Chemistry and Molecular Pharmacology, Harvard Medical School; Howard Hughes Medical Institute, Harvard Medical School

## Abstract

Membrane fusion is an essential step during entry of enveloped viruses into cells. Conventional fusion assays typically report on a large number of fusion events, making it difficult to quantitatively analyze the sequence of the molecular steps involved. We have developed an *in vitro,* two-color fluorescence assay to monitor kinetics of single virus particles fusing with a target bilayer on an essentially fluid support.

Influenza viral particles are incubated with a green lipophilic fluorophore to stain the membrane and a red hydrophilic fluorophore to stain the viral interior. We deposit a ganglioside-containing lipid bilayer on the dextran-functionilized glass surface of a flow cell, incubate the viral particles on the planar bilayer and image the fluorescence of a 100 x 100 μm^2^ area, containing several hundreds of particles, on a CCD camera. By imaging both the red and green fluorescence, we can simultaneously monitor the behavior of the membrane dye (green) and the aqueous content (red) of the particles.

Upon lowering the pH to a value below the fusion pH, the particles will fuse with the membrane. Hemifusion, the merging of the outer leaflet of the viral membrane with the outer leaflet of the target membrane, will be visible as a sudden change in the green fluorescence of a particle. Upon the subsequent fusion of the two remaining distal leaflets a pore will be formed and the red-emitting fluorophore in the viral particle will be released under the target membrane. This event will give rise to a decrease of the red fluorescence of individual particles. Finally, the integrated fluorescence from a pH-sensitive fluorophore that is embedded in the target membrane reports on the exact time of the pH drop.

From the three fluorescence-time traces, all the important events (pH drop, lipid mixing upon hemifusion, content mixing upon pore formation) can now be extracted in a straightforward manner and for every particle individually. By collecting the elapsed times for the various transitions for many individual particles in histograms, we can determine the lifetimes of the corresponding intermediates. Even hidden intermediates that do not have a direct fluorescent observable can be visualized directly from these histograms.

**Figure:**
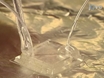


## Protocol

### Glass cover slip functionalization

The planar bilayer used in the fusion assay is supported on a hydrated film of dextran. Dextran acts as a spacer between the planar bilayer and glass surface. This prevents membrane components from becoming stuck on the glass surface and also provides space in to which the contents of a virus particle can escape upon fusion. Glass coverslips are functionalized through treatment with an epoxy silane, which allows us to chemically bond dextran to the glass (Elender, *et al. *1996).

1. Arrange 25 mm coverslips in a ceramic staining rack, and place the rack into a jar or beaker.
2. Fill the container with a 10% solution of laboratory grade glassware detergent. Place the container in an ultrasonic cleaner for 30 minutes.
3. Rinse the container and coverslip rack with deionized water, and refill with 1M postassium hydroxide. Return the container to the bath sonicator for another 30 minutes.
4. Repeat this cleaning procedure using acetone and ethanol.
5. Rinse the coverlips in water and dry.
6. Finally, clean the coverslips with an oxygen plasma stripper for three minutes.
7. The last cleaning step oxidizes the surface, making the glass uniformly hydrophilic and reactive in the following silanization steps.
8. Prepare a 0.2% solution of 3-glycidoxypropyltrimethoxy silane in isopropanol. Immerse the coverslips in this solution for five minutes.
9. Discard the silane solution and rinse the coverslips several times in isopropanol.
10. The coverslips are now coated with a layer of silane, but they must be cured to allow the silane to covalently bond to the glass.  Place the coverslips in an oven set to 80 degrees Celsius for one hour.
11. While the coverslips are curing, weigh out dextran 500 and prepare a 30% w/v solution. Preheating the water will aid dissolution. We will use approximately 1 ml of this solution per coverslip. Once the dextran has dissolved, defoam the solution in a vacuum desiccator.
12. When the silanized coverslips have finished curing, remove them from the ceramic rack and arrange them flat on the inside of a plastic freezer box. Use a 1 ml pipette to cover the top surface of each coverslip with ~ 1 ml of the dextran solution. Close the freezer box and leave in a quiet place for 24-36 hours.
13. While grasping the coverslips with plastic forceps, pour off the excess dextran and replace them into the ceramic rack. Rinse the coverslips several times in water, and allow the coverslips to soak in water for 48 hours. The jar may be gently agitated on a table-top rotator.
14. Dry the coverslips in an oven set to 80 degrees Celsius and store in a vacuum desiccator.

### Liposome Preparation

Supported lipid bilayers are formed by adsorbing liposomes to a dextran functionalized coverslip. The adsorbed liposomes fuse with eachother until membrane ruptures and spreads flat over the surface.

1. A typical liposome formulation used in the assay consists of 1,2,dioleoyl-*sn*-glycero-3-phosphocholine (DOPC), 1-oleoyl-2-palmitoyl-*sn*-glycero-3-phosphocholine (POPC), cholesterol, bovine brain Disialoganglioside (GD_1a_), and N-((6-(biotinoyl)amino)hexanoyl)-1,2-dihexadecanoyl-*sn*-glycero-3-phosphoethanolamine in a ratio of 4:4:2:0.1:5x10^-5^. All components are stored in chloroform or chloroform and methanol solutions.
2. Prepare a mixture containing two micromoles of lipid by transferring volumes of each component into a glass test tube. Evaporate the solvent under a stream of argon or nitrogen gas. Remove the remaining solvent by leaving the test tube in a vacuum desiccator for two hours.
3. Resuspend the dried lipid film in 400 microliters of HNE buffer (5 mM HEPES, 145 mM NaCl, 0.1 mM EDTA).
4. Transfer the suspension to a plastic microcentrifuge tube. Freeze the suspension in a liquid nitrogen bath and thaw in a warm water bath. Repeat this freeze/thaw cycle four times.
5. Assemble a lipid extruder with a 100 nm polycarbonate membrane filter, and extrude the lipid suspension 25 times. The suspension should appear to be more transparent after extrusion.
6. Liposomes should be used on the day they are prepared. They may be stored at room temperature until used.

### Microfluidic flow cell preparation

A simple microfluidic flow cell is made by sandwiching double stick tape between a quartz slide and a functionalized coverslip.

1. Obtain a 20x20x3 mm quartz slide. Use a diamond burr to drill two 1 mm holes on opposite sides.
2. Cut a 20 mm square from a sheet of double stick tape, and using a razor blade, cut out a 15 x 2 mm section from the center.
3. Peel off one side of the tape backing, and adhere the tape to the quartz slide. Adhere the other side to a functionalized coverslip.
4. Cut two 20 cm  lengths of polyethylene tubing and insert them into the holes in the quartz slide.
5. Seal the flow cell with 5 minute epoxy glue.
6. When the glue has cured, prime the flow cell and tubing with buffer.
7. A supported lipid bilayer can be formed at this stage by drawing approximately 80 microliters of the liposome suspension into the channels.

### Virus particle labeling

1. H3N2 influenza A (X-31) is typically used for the fusion assay, but other flu strains and other viruses have been used successfully.
2. Virus is labeled with up to two different fluorescent dyes to allow detection of hemifusion and pore-formation.
3. Dissolve sulforhodamine b (SRB) in HNE buffer to make a 20 mM solution. Combine 20 microliters of the dye solution with 10 microliters of the viral suspension, and leave at room temperature for 20 hours. During this period, dye will slowly accumulate inside the virus particles.
4. Remove the excess dye by passing the the virus suspension through a PD-10 desalting column. If enough viral material is used, a colored band can be seen in the column. This band contains labeled virus and can be collected as it is eluted.
5. If no band is visible, collect 200 microliter fractions and determine which fractions contain labeled virus by measuring 565 nm absorption on a spectrophotometer. The virus should elute in a total volume of approximately 0.8 ml.
6. Label the viral envelope by adding 13 microliters of a 2 mM dimethyl formamide (DMF) solution of octadecyl rhodamine 110 (Rh110C18) to the SRB labeled virus suspension.
7. Stir the suspension by attaching the tube to a laboratory rotator and leave for 3 hours.
8. Purify the virus from excess Rh110C18 using a PD-10 desalting column as described before.

### Microscope configuration

The experiment is performed on a fluorescence microscope equipped with a high NA oil immersion objective suitable for total internal reflection fluorescence microscopy. The 488 and 568 nm lines from an argon/krypton gas laser are used to excite the Rh110C18 and SRB labeled virus. Simultaneous imaging of each label is accomplished by splitting the green and red fluorescence emission with a dichroic mirror and independently focusing the images onto separate halves of a electron multiplying CCD camera.

### Performing the Assay

Execution of the fusion assay involves a stepwise assembly of the biochemical components within the flow cell: assembly of the planar lipid bilayer, docking of labeled virus particles, coating the surface with fluorescein, and initiation of the reaction with low pH buffer (Figure 1A).

1. Mount the flow-cell onto the microscope stage, and attach the outlet tubing to a syringe pump set to flow at a rate of 40 microliters per minute.
2. Clear the flow channels of excess liposomes by flowing in 300 microliters of HNE.
3. Focus the microscope and adjust the laser power to illuminate the surface with 350 W/cm^2^ of 488 nm light and 70 W/cm^2^ of 568 nm light. If you are using a 1.45 NA 60x oil immersion objective, set the laser intensities to 100 micro Watts and 20 micro Watts, respectively.
4. Pump labeled virus (diluted 1/100) into a flow cell. As the virus diffuses to the bottom surface of the flow channel, particles will begin to stick to the ganglioside incorporated in the  lipid bilayer.
5. When a suitable density of docked virus particles has been attained (up to around 1000 particles per field of view). Switch the inlet tubing to a solution containing two micrograms per milliliter fluorescein labeled streptavidin. The labeled streptavidin will bind to biotin coupled lipid molecules in the bilayer, and eventually a dim green background will be visible.
6. Wash the flow channel with 300 microliters of HNE.
7. Choose an imaging area, focus the microscope and prepare the CCD camera for time-lapsed image acquisition. Set the exposure time to 100 ms and collect frames at a rate of 10 Hz.
8. Initiate fusion by flowing in an acidic buffer containing 10 mM sodium citrate and 140 mM NaCl. The pH of the buffer should be adjusted to be below the critical pH of the virus being assayed. X-31 fuses at pH’s below 5.5.

### Representative Results

Figure 1B shows snapshots of bilayer docked virions before and during fusion. Each diffraction limited spot represents an individual virus particle. Red and green fluorescence have been separately imaged onto the top and bottom halves of a CCD camera, allowing simultaneous observation of the viral envelope and content labels. There are typically twice as many spots in the green channel compared to the red, and this reflects the lower efficiency of labeling by the content dye compared to the envelope label. The green background fluorescence emitted from surface bound fluorescein disappears upon arrival of the citrate buffer and allows determination of the start time of the fusion reaction. Hemifusion events are detected as bursts of green fluorescence as the quenched Rh110C18 in the viral envelope diffuses across the hemifusion stalk and is diluted in the planar membrane. An outward expanding fluorescent cloud can be seen around hemifused particles, demonstrating free diffusion of the fatty-acyl linked dye in the planar membrane. Pore formation is observed as the decay of red fluorescence from SRB trapped within the interior of the viral particles. The opening of a fusion pore allows the dye to escape into the aqueous space below the planar bilayer and out of the observation region.

Fluorescence intensity trajectories for each particle, plotted from the integrated intensities of individual particles, facilitate determination of the yield and times of hemifusion and pore formation (Fig. 1C). Hemifusion and pore formation event times are determined as the point at which the maximum slope of a Rh110C18 fluorescence burst or SRB signal decay occurs. In a successful experiment, about 50 percent of the particles labeled with Rh110C18 yield dequenching signals, and 30 percent of the SRB labeled particles give useable signal. Of the particles labeled with both dyes, approximately 10 percent show both lipid mixing and pore formation signals.

Fig. 2A-C shows the distribution of hemifusion and pore formation lag times from a typical experiment. Fig.2D-F shows event distributions of events combined from five experiments performed under the same conditions. Even with a modest number of observations, the shape of the histograms is apparent. Because each experiment is synchronized by detection of the pH drop time, multiple experiments can be combined and detailed information about rate limiting intermediate steps can be obtained.


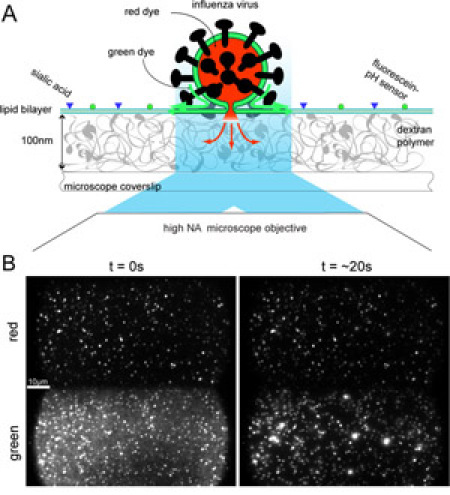
 
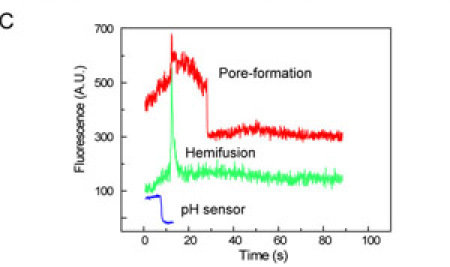
 **Figure 1.** Experimental design. A) Virus particles are labeled with two fluorescent dyes to monitor the kinetics of hemifusion and fusion pore formation. Fluorescence is collected by a high-NA microscope objective and imaged onto a CCD. B) Fluorescence images before (left) and during (right) the fusion of individual viral particles. Top and bottom half of each image correspond to the red and green fluorescence, respectively, of the same ~ 50 x 100 mm^2^ area of the supported bilayer. C) The fluorescence intensity of the red SRB viral content tracer (upper trace), the green Rh110C18 membrane dye (middle trace), and the fluorescein pH sensor (lower trace) provide exact times elapsed between pH drop, hemifusion, and fusion. Please click here to see a larger version of figure 1.


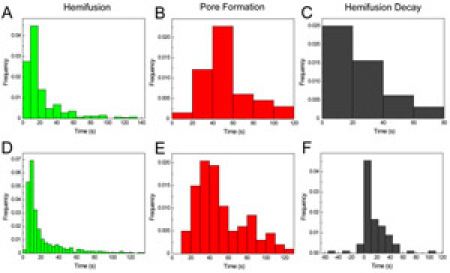
 **Figure 2.** Fusion kinetics of fluorescently labeled virus. Distributions of the time elapsed between the pH drop and hemifusion (A, D) and pore formation (B,E). The rise and decay of the distributions indicate the presence of multiple intermediate steps. The transition between hemifusion and opening of a fusion pore for individual particles is exponentially distributed, suggesting a single rate-limiting step. Panels A-C show the results of a single experiment. Panels D-F are compiled from five separate experiments performed under the same conditions. Please click here to see a larger version of figure 2.

## Discussion

Preparation of fluid and continuous supported lipid bilayers can be challenging. Trace amounts of contaminating material or surface defects will prevent spreading of bilayers. Careful cleaning and deposition of a uniform layer of dextran are essential.

Prolonged imaging of virus particles can bleach the fluorescent labels or cause them to become inactivated. Photo-damage of this kind is well known in the bio-imaging and single molecule fields and is generally believed to stem from the generation of reactive oxygen species by the excited fluorophores. To minimize photo-damage, we generally minimize the excitation laser power and avoid prolonged exposure prior to starting the experiment. Depletion of oxygen from the buffers with one of the several oxygen scavenging systems reported may prove useful.

The ability to get information about transient intermediate states requires that the fusion reaction be precisely synchronized. The flow rate used and the dead volume of the inlet tubing and flow cell channel are factors that can limit synchronization. Fusion is initiated by exchanging neutral for acidic pH buffer. However, as the acidic buffer travels through the inlet tubing into the flow cell, the interface between the two buffers mixes through diffusion and becomes progressively less well defined. The resulting pH gradient at the bilayer surface tends to blur determination of the start time. Furthermore, since fusion kinetics is dependent on proton concentration, a pH gradient at the start of the experiment could complicate interpretation of the results. In the experiment demonstrated here, the kinetics of fusion is much slower than the pH transition time, and the gradient effect does not significantly affect fusion kinetics. We are currently working on modifications to our flow cells that would allow us to prime the inlet tubing with the desired buffer before diverting the flow into the flow cell.
